# *Staphylococcus aureus*-Induced G2/M Phase Transition Delay in Host Epithelial Cells Increases Bacterial Infective Efficiency

**DOI:** 10.1371/journal.pone.0063279

**Published:** 2013-05-23

**Authors:** Ludmila Alekseeva, Lucie Rault, Sintia Almeida, Patrick Legembre, Valérie Edmond, Vasco Azevedo, Anderson Miyoshi, Sergine Even, Frédéric Taieb, Yannick Arlot-Bonnemains, Yves Le Loir, Nadia Berkova

**Affiliations:** 1 Shemyakin-Ovchinnikov Institute of Bioorganic Chemistry, Moscow, Russian Federation; 2 Institut National de la Recherche Agronomique, Unité Mixte de Recherche 1253, Science et Technologie du Lait et de l'Œuf, Rennes, France; 3 AGROCAMPUS OUEST, Unité Mixte de Recherche 1253, Science et Technologie du Lait et de l'Œuf, Rennes, France; 4 Universidade Federal de Minas Gerais, Instituto de Ciencias Biologicas, Departamento de Biologia Geral, Belo Horizonte, Minas Gerais, Brazil; 5 Institut de Recherche en Santé, Environnement et Travail, U1085, Université Rennes-1, Rennes, France; 6 Institut National de la Recherche Agronomique, USC U1043, Institut National de la Santé et de la Recherche Médicale, Toulouse, France; 7 CNRS, Unité Mixte de Recherche 6290, Biologie, Santé, Innovation technologique, Université Rennes-1, Rennes, France; Aarhus University, Denmark

## Abstract

*Staphylococcus aureus* is a highly versatile, opportunistic pathogen and the etiological agent of a wide range of infections in humans and warm-blooded animals. The epithelial surface is its principal site of colonization and infection. In this work, we investigated the cytopathic effect of *S. aureus* strains from human and animal origins and their ability to affect the host cell cycle in human HeLa and bovine MAC-T epithelial cell lines. *S. aureus* invasion slowed down cell proliferation and induced a cytopathic effect, resulting in the enlargement of host cells. A dramatic decrease in the number of mitotic cells was observed in the infected cultures. Flow cytometry analysis revealed an *S. aureus*-induced delay in the G2/M phase transition in synchronous HeLa cells. This delay required the presence of live *S. aureus* since the addition of the heat-killed bacteria did not alter the cell cycle. The results of Western blot experiments showed that the G2/M transition delay was associated with the accumulation of inactive cyclin-dependent kinase Cdk1, a key inducer of mitosis entry, and with the accumulation of unphosphorylated histone H3, which was correlated with a reduction of the mitotic cell number. Analysis of *S. aureus* proliferation in asynchronous, G1- and G2-phase-enriched HeLa cells showed that the G2 phase was preferential for bacterial infective efficiency, suggesting that the G2 phase delay may be used by *S. aureus* for propagation within the host. Taken together, our results divulge the potential of *S. aureus* in the subversion of key cellular processes such as cell cycle progression, and shed light on the biological significance of *S. aureus*-induced host cell cycle alteration.

## Introduction

*Staphylococcus aureus* is a highly versatile Gram-positive pathogen that can cause life-threatening infections such as bacteremia, pneumonia, osteomyelitis, meningitis, endocarditis and sepsis [1]–[3]. Staphylococcal infection is also a serious concern in animal health. Notably, it is a major cause of mastitis in ruminants for which existing prevention or treatment strategies are often inefficient [4].

Epithelial cells are able to sense microbes, creating an early line of defense against pathogens [5]. Colonization of the host tissue by *S. aureus* is attributed in part to its capacity to adhere to the epithelial cells, the first step of infection, and to the production of bacterial toxins that lead to immune evasion [6], [7]. Several studies have reported that *S. aureus* can be internalized within the host epithelial cells and may therefore contribute to persistent infections [8].

Pathogens have highly sophisticated mechanisms to hijack the main function of the host cells, thus promoting their invasion and colonization. These effects include induction of membrane ruffling, alteration of host cell apoptosis, promotion of cell proliferation and, conversely, inhibition of cell growth [9]–[11]. In the last decade, special attention has been given to the growing family of bacterial cyclomodulins that alter the eukaryotic cell cycle [12]. This cycle consists of the G1 phase characterized by cell growth, the S phase characterized by DNA replication, the G2 phase in which cells are prepared for division, the M phase during which mitosis occurs, and the G0 phase when cells can enter a quiescent state. *Escherichia coli* cycle-inhibiting factor (Cif) induces G2 arrest of the host cell cycle [13]. *Bacillus anthracis* edema toxin and *Bordetella pertussis* adenylate cyclase toxin increase the proportion of cells in the G1/G0 phase. [14]. Exposure of eukaryotic cells to the cytolethal distending toxin of *E. coli* results in arrest in both the G1 and G2 phases [12], [15]. Pathogen-induced cell cycle alteration may be linked to the inhibition of cyclin-dependent kinases (CDKs), key effectors responsible for cell cycle progression [16], as well as to the post-translational modifications of histones, nuclear proteins that package DNA into nucleosomes, the chromatin units whose functions are related to the prevention of DNA damage and the control of gene expression and DNA replication [17]. Few studies have focused on the relationship between exposure of eukaryotic cells to *S. aureus* and the host cell cycle. *S. aureus* is involved in the activation and differentiation of resting B cells [18]. *S. aureus* epidermal cell differentiation inhibitor affects the differentiation of cultured keratinocytes [19]. Exposure of keratinocytes to staphylococcal alpha-toxin resulted in the doubling of the S+G2/M phase [20]. Transcriptome analyses of the human corneal epithelial cells exposed to *S. aureus* and *S. aureus*-infected bovine mammary tissue revealed an alteration in the expression profiles of genes that affect the cell cycle progression [21], [22]. To our knowledge, the implications of *S. aureus* in the alteration of the eukaryotic cell cycle and the biological significance of such an alteration has never been investigated.

In the present study, we demonstrated a G2/M phase transition delay in *S. aureus*-infected epithelial cells. Analysis of the cell cycle alteration revealed accumulation of the inactive cyclin-dependent kinase, Cdk1, and the accumulation of unphosphorylated histone, H3, which was correlated with a reduction of the mitotic cell number. As far as we know, this is the first study showing the G2/M transition delay of epithelial cells following invasion by *S. aureus* bacterial cells. Analysis of *S. aureus* replication suggested that the G2 phase was preferential for its infective efficiency.

## Materials and Methods

### Eukaryotic cells and growth conditions

Since *S. aureus* is the leading cause of superficial infections in humans and animals, two types of epithelial cells representative of human and animal hosts were used in this study. Human cervix cancer HeLa cell line was cultured in cDMEM (Dulbecco's modified Eagle medium, GlutaMax, 10% fetal calf serum (FCS)) supplemented with 100 U/mL penicillin, and 100 µg/mL streptomycin sulfate (Gibco BRL, Cergy Pontoise, France) up to 80% confluence at 37°C with 5% CO_2._ The bovine mammary epithelial cell line (MAC-T) [23] was provided by Nexia Biotechnologies (Quebec, Canada). MAC-T cells were grown in cDMEM supplemented with 5 µg/mL insulin, 5 µg/mL hydrocortisone (Sigma, St. Quentin Fallavier, France), 100 U/mL penicillin and 100 µg/mL streptomycin sulfate. Trypsin/EDTA (Invitrogen, Saint Aubin, France) was used to release adherent MAC-T and HeLa cells for subculturing.

### Bacterial strains and growth conditions

We previously showed that *S. aureus* strains of human and animal origins had different genomic profiles [24] that may reflect host-specificity [25]. Three *S. aureus* strains of human and ruminant origin were thus used in this study. The methicillin-resistant *S. aureus* MW2 strain was selected as a representative strain of human origin. The O46 and O11 strains were chosen as being representative of well-characterized animal strains and were shown to induce subclinical (O46) and lethal gangrenous mastitis (O11) in a ewe model [26]–[28]. All cultures were performed as follows: aliquots from overnight cultures on Brain Heart Infusion (BHI) broth were diluted (1∶50) in DMEM. *S. aureus* strains were grown in tubes (50 mL) and incubated at 37°C under anaerobic conditions until cultures had reached an optical density of 0.6 at 600 nm, corresponding to approximately 10^8^ CFU/mL (CFU, colony-forming unit) [29]. The staphylococci were harvested by centrifugation, washed twice with phosphate-buffered saline (PBS), and resuspended in the interaction medium (DMEM). Bacterial concentrations were estimated spectrophotometrically and the number of live bacterial cells was confirmed by plate counts. To prepare heat-killed bacteria, broth-containing tubes (total volume: 3 ml) were placed in an 80°C water bath for 30 min, as previously described [30]. The staphylococci were centrifuged, washed with PBS, and resuspended in DMEM. To verify the absence of viable bacterial cells, a 100-µl portion of heat-treated culture was surface-plated in duplicate onto BHI agar and incubated at 37°C for 24 h. No colony was detected when heat-treated bacteria were plated onto BHI agar.

### Cell synchronization: double thymidine block

For cell synchronization, we adapted the double thymidine block (DTB) protocol previously described: the cells were synchronized at the G1/S border [31], [32]. Briefly, HeLa or MAC-T cells were grown either in a 25-mL flask, in 96-well plates, or on cover slips up to 30% confluence. After washing with PBS, the cells were cultivated in cDMEM containing 2 mM thymidine (cDMEM-T) for 18 h. Thymidine was then removed by washing with PBS and the cells were cultivated in fresh cDMEM for 9 h to release cells. The cells were then cultivated in cDMEM-T for 17 h, followed by cultivation in fresh cDMEM.

### Cell culture infection

Eukaryotic cells (at 30% confluence at the beginning of DTB) were infected with *S. aureus* with a multiplicity of infections, MOIs (number of bacteria per cell at the onset of infection) ranging from 5∶1 to 20∶1 in DMEM at the periods indicated after DTB release. Cell concentrations at the time bacteria were added or at the periods indicated were determined using one of the four samples prepared for each MOI. The remaining samples were used for the analysis in triplicate. For the microscopic evaluation, the cells were grown overnight on cover slips (Marienfield, Lauda-Königshofen, Germany) in 12-well plates (Nunc, NuclonTM Surface, Thermo Scientific, Langenselbold, Germany). Bacterial concentrations were estimated spectrophotometrically and were confirmed by plate counts. The low cell density (30% confluence) at the beginning of the experiment was used to ensure cell proliferation during the entire experiment since cells cease to proliferate at high cell density and enter a viable state of quiescence, as previously reported [33] (and on the basis of our observations). An MOI value was selected based on preliminary experiments since high concentrations of bacteria induced cell death. Unbound bacteria were removed 2 h post-infection by washing the wells with PBS, followed by incubation in cDMEM containing 100 μg/mL gentamicin (cDMEM-Gent100) for 2 h, which eliminates the extracellular bacteria and retains the intracellular bacteria [34], followed by incubation in cDMEM containing 25 μg/mL of gentamicin (cDMEM-Gent25) for the periods indicated. The incubation time was chosen according to the effects investigated, in agreement with the recognized evaluation of the phases of the HeLa cell cycle and on the basis of the preliminary experiments, which made it possible to observe the significant difference between the control and infected cell cultures.

To test the specificity of the *S. aureus* effect, some cell cultures received 2-µm latex beads instead (25 beads/cell). The effect of live *S. aureus* bacteria was also compared to that of heat-killed bacteria used at the same concentrations.

Cell morphology was examined by phase-contrast microscopy. The nuclei were stained with DAPI. To evaluate the cell size, the cell area was measured using the merged images of phase contrast and DAPI-stained cells with ImageJ software. A region was drawn around each cell to be measured. An area of at least 500 cells was measured for each condition. The results were presented using the arbitrary unit of the area. All the experiments were performed at least three times.

### Cell proliferation assay

HeLa (3.5×10^3^) or MAC-T (4×10^3^) cells were incubated in 96-well plates for 24 h and were then exposed to *S. aureus* at MOIs of 5∶1, 10∶1 and 20∶1 for 2 h. The initial cell concentrations were selected on the basis of the preliminary experiments to ensure that the cells still proliferate at the end of the cell-bacteria co-incubation period. Unbound bacteria were removed 2 h post-infection by washing the wells with PBS, followed by incubation in cDMEM-Gent100 for 2 h, and then by incubation in cDMEM-Gent25 for 24 h, 48 h and 72 h. The number of cells was determined with a hemacytometer after the indicated periods of infection. Viability of *S. aureus*-infected cells was measured using the colorimetric MTT [3-(4,5-dimethylthiazol-2-yl)2,5-diphenyltetrazolium bromide] assay Tox-1 kit (Sigma, Saint-Quentin Fallavier, France) according to the manufacturer's recommendations. Absorbance was measured at 570 nm with a reference filter of 630 nm using a plate reader (Molecular Devices, Gregoire, France). The ratio of living cells was calculated as described in Orosz et al. [35]. The absorbance values of the negative control of cells cultured in medium only were arbitrarily set at 100% cell viability. The experiments were performed three times.

### Analysis of cytotoxic effects of *S. aureus*

MAC-T and HeLa cells were cultivated in 96-well plates and exposed to *S. aureus* as described above. At the indicated time interval, attached and floating cells were harvested and washed in cold PBS. The viability in control and *S. aureus*-treated cultures was estimated by cell counts using a hemacytometer combined with the trypan blue exclusion assay. The results were calculated as the percentage of live cells out of the total number of cells. The experiments were performed three times.

### Fluorescence microscopy: mitotic index evaluation and immunofluorescence

Mitotic index evaluation was performed with the cells grown on the cover slips as described [36]. Briefly, HeLa or MAC-T cells (2×10^5^ cells) were grown overnight on cover slips (Marienfield, Lauda-Königshofen, Germany) in 12-well plates (Nunc, NuclonTM Surface, Thermo Scientific, Langenselbold, Germany) in triplicate up to 30% confluence, followed by DTB synchronization. Four hours after the second block release, cells were exposed to human MW2 or ovine O46 strains for 2 h, at MOIs ranging from 5∶1 to 20∶1. Two hours post-infection, unbound bacteria were removed by washing the wells with PBS, followed by incubation in cDMEM-Gent100 for 2 h. The cells were then incubated in cDMEM-Gent25 for 25 h. Plates were subsequently centrifuged to avoid loss of mitotic cells during washing, and the cells were then fixed with 4% PFA (4% -weight/volume-paraformaldehyde dissolved in PBS) for 1 h. The cover slips were then mounted on slides with DAPI-containing ProLong antifade Vectashield medium (Vector Laboratory, Les Ulis, France). The mitotic indexes (number of cells in mitosis vs. total number of cells) in infected synchronous cell culture were compared to the mitotic indexes in uninfected synchronous HeLa cells at the same time point, as well as to the control assynchronous HeLa cells. Mitotic cells were microscopically distinguished from interphase cells. The same number of cells, 400 cells per assay, was counted with a Nikon fluorescence microscope using ×400 magnification.

The method of mitotic index analysis of the cells that were grown on the cover slips [36] was compared to the method where the cells were grown in the wells, collected and fixed in a solution containing 4%PFA, 0.5% NP-40 and Hoescht 33258 in PBS [37]. Nuclei were visualized by fluorescent microscopy. There was no significant difference between these two methods (data not shown). The method of mitotic index evaluation using the cells grown on the cover slips was chosen for the study.

To stain phosphorylated H3 histone (p-Ser10 Histone H3), synchronized HeLa cells were exposed to the MW2 strain (MOI 20∶1) and immunofluorescence was performed as described [38]. After 4% PFA fixation, cells were permeabilized with 0.3% Triton X-100/PBS solution and then incubated in 10% goat serum (Sigma) for 1 h at room temperature. Cells were then incubated overnight with rabbit anti-p-Ser10 Histone H3 antibody (Millipore, Molsheim, France) (1∶1000 in 3% BSA/PBS), followed by incubation with goat anti-rabbit FITC-labeled antibody (Sigma) at a dilution of 1∶2000 for 2 h at room temperature. After washing, the cover slips were mounted on slides with ProLong antifade Vectashield medium containing DAPI, as described above. Samples were viewed with a Nikon fluorescence microscope (using ×40 and ×400 magnifications) coupled with a camera. Cells incubated only with secondary antibody as a negative control showed no reactivity. The images were captured with strictly identical acquisition settings for every sample. Fifty mitotic cells in infected vs. non-infected culture were analyzed. The relative level of fluorescence (corrected total cell fluorescence (CTCF) arbitrary unit) of p-Ser10 Histone H3-positive mitotic nuclei was measured using ImageJ software, as previously described [39].

A region was drawn around each cell to be measured. The same region in an area without fluorescent cells was used for background subtraction. The following formula was applied:

CTCF  =  Integrated Density – (area of selected cell × mean fluorescence of background readings).

To visualize the intracellular bacteria in some experiments, *S. aureus* bacteria were stained with SYTO 9 dye using LIVE/DEAD BacLightTM bacterial viability kit (Invitrogen, USA). MAC-T cells were grown overnight on cover slips and were infected with SYTO 9-stained bacteria. Two hours post-infection, unbound bacteria were removed by washing the wells with PBS, followed by incubation in cDMEM-Gent100 for 2 h. Plates were subsequently centrifuged and the cells were then fixed with 4% PFA for 1 h. The cover slips were then mounted on slides with ProLong antifade Vectashield medium containing DAPI (Vector Laboratory, Les Ulis, France). The cells were then observed under a fluorescence microscope. The bacteria fluoresce green.

### Flow cytometry analysis (FACS)

HeLa cells (5×10^4^ cells) were grown in 25-mL flasks up to 30% confluence, followed by DTB synchronization. Four or six hours after the second thymidine release, cells were exposed to *S. aureus* MW2 for 2 h. Wells were washed with PBS 2 h post-infection and incubated in cDMEM-Gent100 for 2 h, followed by incubation in cDMEM-Gent25 for the periods indicated. Detached cells were then combined with adherent cells, washed with PBS, and fixed in 70% ethanol overnight. Fixed cells were then washed in PBS and stained with propidium iodide (PI) (20 mg/mL) in PBS containing 0.2 mg/mL DNase-free RNase A for 30 min at 37°C in the dark [14]. Cells were analyzed by FACS (FACSCalibur, Becton Dickinson, Le Pont de Claix, France) using an excitation wavelength of 488 nm and emission at 585 nm. Data were collected from 20,000 cells and analysis was performed with Cell Quest software. Each experiment was performed at least four times. Preliminary experiments were carried out to verify the influence of gentamicin on the cell cycle distribution. There were no differences between cell cycle progression in the cell cultures containing gentamicin and those that did not.

### Semi-quantitative Western blot analysis

HeLa cells (5×10^4^) were grown and synchronized as described for FACS experiments. Six hours after DTB release, cells were exposed to *S. aureus* MW2 for 2 h. Wells were washed with PBS 2 h post-infection and incubated in cDMEM-Gent100 for 2 h, followed by incubation in cDMEM-Gent25 for 20 h. Cells were then suspended in 100 μL 1X Laemmli loading buffer [40], sonicated for 5 seconds, heated for 5 min at 100°C, and then separated on 12% SDS polyacrylamide gels, as previously described [32]. Proteins were subsequently transferred onto a PVDF membrane. Membranes were blocked for 1 h at room temperature in Tris-buffered saline containing 0.025% Tween-20 (TBS-T) with 5% nonfat dry milk and probed with 1∶1000 anti-phospho-Cdk1 antibodies (Cell Signaling Technology, Saint Quentin Yveline, France), followed by incubation with horseradish peroxidase-conjugated secondary antibodies (1∶1000 dilution in TBS-T). Bands were visualized with an enhanced chemiluminescence (ECL) detection kit (Pierce, Illkirch, France). The membrane was re-probed with anti-actin antibody (Sigma) to assess protein loading [32]. To study histone phosphorylation, a PVDF membrane was incubated with anti-p-Ser10 Histone H3 antibody (1∶1000), followed by ECL detection. The membrane was then treated twice with stripping buffer (50 mM Tris-HCl (pH 6.7), 2% SDS, and 200 mM β-mercaptoethanol) at 60°C for 30 minutes, washed with TBS-T, blocked for 1 h with TBS-T in 5% nonfat dry milk solution and re-probed with anti-total histone H3 antibodies (Millipore, Molsheim, France) to assess the total level of histone H3. The chemiluminescence reaction was visualized and processed using a G:BOX imaging system (Syngene, Ozyme, Poitiers, France). To carry out a relative quantification of samples, densitometric analysis of Western blots (a method for measuring the intensity of a band in a gray-scale image) was performed using Gene Tools software (Syngene). Band intensities in the linear range of the signal were measured using underexposed membranes. Two different concentrations of each sample were tested during the same Western blot analysis, making it possible to verify the proportionality of the band intensities. The samples with lower concentrations were used for densitometric analysis. One of the samples was used in all experiments as a reference for densitometric analysis.

Data are presented as mean ± SD from three densitometry scans, adjusted with total histone H3, and expressed as fold changes over the appropriate control, as previously described [17].

### Analysis of the bacterial population in HeLa cells synchronized in G1 or G2 cell cycle phases

CDK4/6 inhibitor IV (Calbiochem, Darmstadt, Germany), which prevents G1/S transition [41], was used to enrich cell culture with cells in the G1 phase. Reversible CDK1 inhibitor, RO-3306 (Calbiochem), was used to arrest cells in the G2 phase [42]. Cells were maintained in the inhibitor-containing medium throughout infection. HeLa cells (10^5^ per well in 12-well plates) were grown overnight and then incubated in cDMEM containing either 10 µM CDK4/6 inhibitor IV or 9 µM of RO-3306 for 3 h. *S. aureus* at MOI 10∶1 and 50∶1 was then added to the cells and incubated for 2 h (T0). Cell concentrations at the time that bacteria were added or at the periods indicated were determined using one of the four samples. The remaining samples were used for the analysis in triplicate. Following washing with PBS, cells were incubated in cDMEM-Gent100 containing inhibitors for an additional 2 h (T2), which makes it possible to remove the extracellular bacteria and retain the intracellular bacteria (internalization assay) [8].

The cells then were incubated in cDMEM-Gent25 containing inhibitors for 4 h (T4) or 20 h (T20). The incubation time was determined according to previous studies that showed the intracellular replication of *S. aureus* at 4 h and 20 h post-infection in various cell lines [8], [43]. At T0, T2, T4 and T20, cells (adherent and obtained from the supernatants) were counted using a hemacytometer by trypan blue exclusion assay. After lysis with 0.05% Triton X-100 in PBS, cell lysates were plated on BHI agar, and CFU were determined after overnight incubation. To compare the number of intracellular bacteria in G1 or G2 phase-arrested cells with the number of bacteria in the asynchronous cells, data were expressed as CFU values normalized to 10^5^ HeLa cells. The proportions of cells in the different cell cycle stages at T0, T2, T4 and T20 were determined by FACS.

### Statistical analysis

At least three different assays were performed per experiment. The differences among the groups were assessed by analysis of variance (ANOVA). P-values <0.05 were considered to be significant. Tukey's Honestly Significant Difference test was applied for comparison of means between the groups. The values are expressed as mean ± standard deviation (±SD).

## Results

### Exposure of epithelial cells to *S. aureus* resulted in the enlargement of the host cells

Invasion of host cells by bacterial pathogens such as certain *E. coli* or *Salmonella typhimurium* strains reportedly results in changes in cell morphology characterized by a progressive enlargement of the cell body and nucleus and by the absence of mitosis [32], [44].

**Figure 1:**
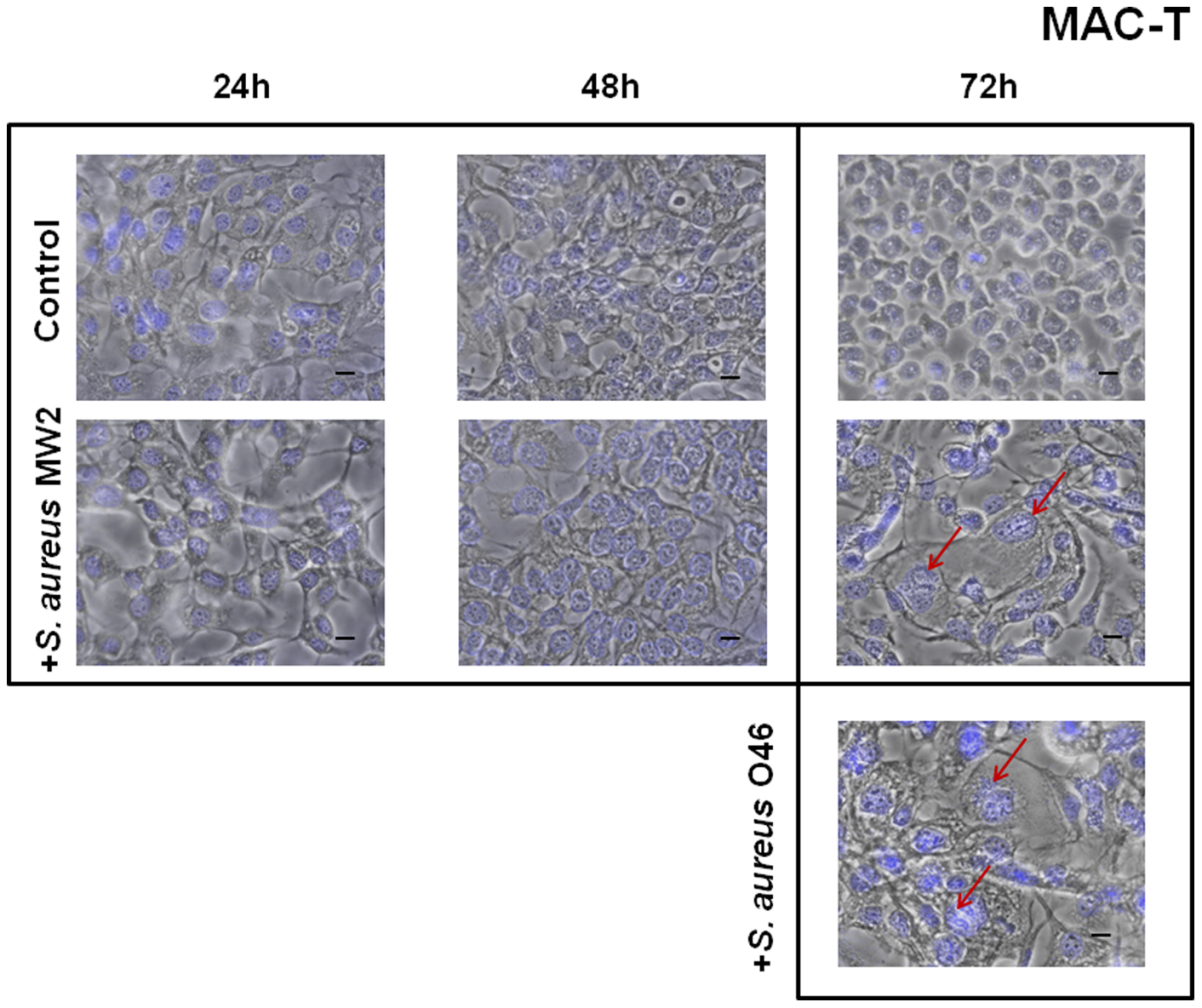
Enlargement of MAC-T cells exposed to *S. aureus*. Bovine MAC-T cells were exposed for 2 h to *S. aureus* MW2 or O46 MOI 20∶1, **treated with gentamicin for 2** **h** and further incubated for 24 h, 48 h and 72 h. After incubation, the cells were fixed, stained with DAPI and observed using ×400 magnification. The merged image of phase contrast and DAPI-stained cells is presented. Red arrows indicate the enlarged cells in infected cell cultures. Scale bars: 10 µm. A. MAC-T cells exposed to *S. aureus* MW2 for 24 h, 48 h and 72 h at MOI 20∶1. B. MAC-T cells exposed to *S. aureus* O46 for 72 h at MOI 20∶1.

**Figure 2:**
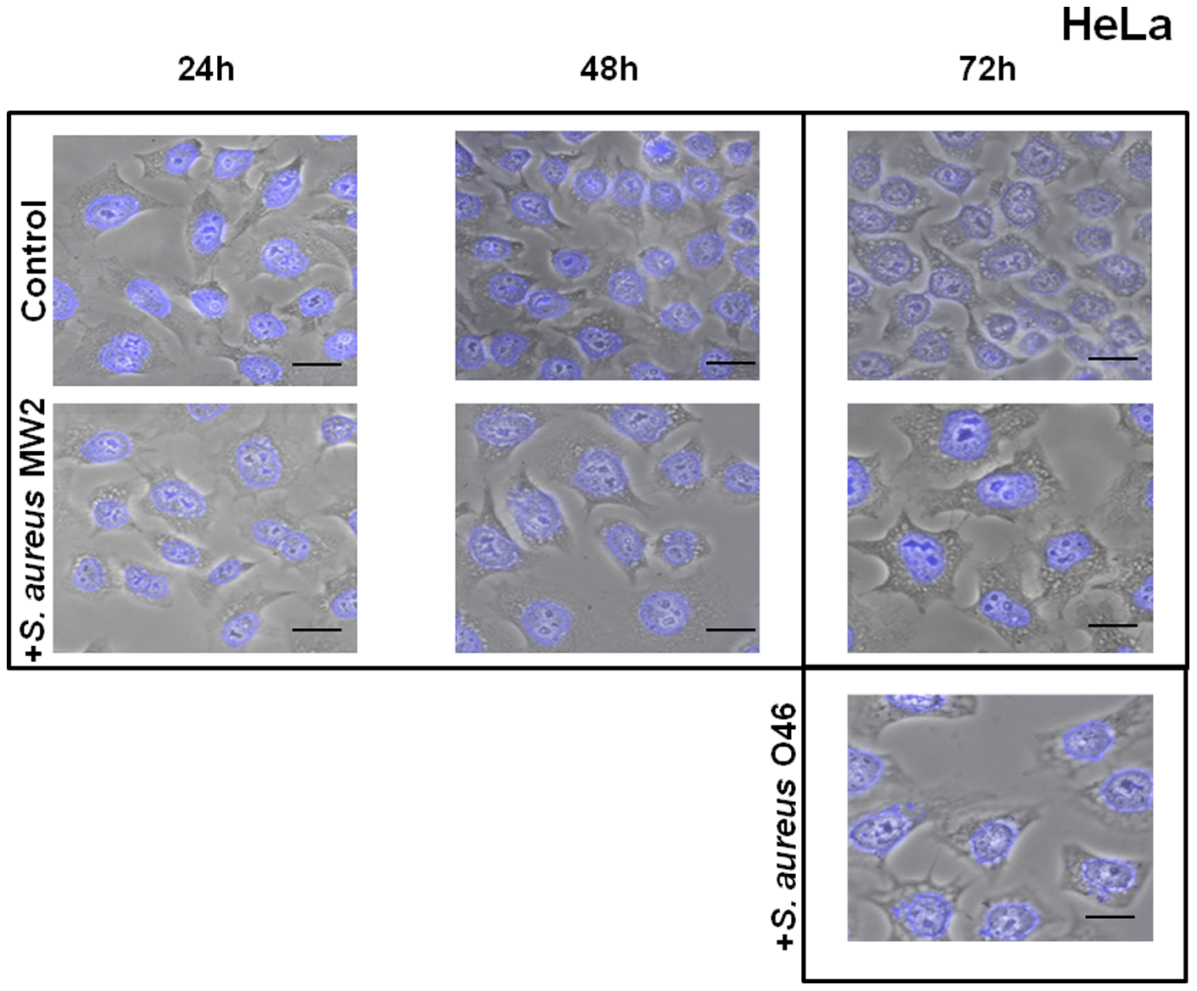
Enlargement of HeLa cells exposed to *S. aureus*. Human HeLa cells were exposed for 2 h to *S. aureus* MW2 or O46 at MOI 20∶1 and further incubated for 24 h, 48 h and 72 h. After incubation, the cells were fixed, stained with DAPI and observed using ×400 magnification. The merged image of phase contrast and DAPI-stained cells is presented. Red arrows indicate the enlarged cells in infected cell cultures. Scale bars: 10 µm. A. Cells exposed to *S. aureus* MW2 for 24 h, 48 h and 72 h at MOI 20∶1. B. Cells exposed to *S. aureus* O46 for 72 h at MOI 20∶1.

In this study, host cell morphology was examined microscopically after 2 h incubation with *S. aureus* at MOI 20∶1 and at various time intervals (24 h, 48 h and 72 h) after removal of extracellular *S. aureus* cells by a gentamicin treatment (see Materials and Methods for details). To visualize the intracellular bacteria, SYTO 9-stained *S. aureus* bacteria were observed after 2 h of cDMEM-Gent100 treatment under a fluorescent microscope in the preliminary experiments (**Fig. S1**). Both strains (MW2 human isolate and O46 ruminant isolate) were tested on bovine MAC-T (**Figs. 1**) and human HeLa (**Figs. 2**) cell lines. No difference was detected between the control cells (subjected to gentamicin treatment but not to *S. aureus* invasion) and the infected cells at 24 h and 48 h **of the further incubation**, whereas a cytopathic effect of *S. aureus,* characterized by cell enlargement, was observed 72 h post-infection: both strains induced analogous cytopathic effects, and cell and nuclei sizes in infected MAC-T cells (red arrow) were bigger than those of the control cells (**Fig. 1**). This enlargement of the nuclei may result from cell cycle alteration, as previously shown for the cytopathic effect of colibactin, a genotoxin produced by some *E. coli* strains [32]. The cytopathic effect of *S. aureus* was further investigated on human HeLa cells. These cells are well characterized and are commonly used to study pathogen-induced cytopathic effects and cell cycle alteration [32], [45]. Both *S. aureus* strains, regardless of their human or animal origin, induced analogous cytopathic effects on the HeLa cells: the cell size and DAPI-stained nuclei of infected cells were twice as big as those of the control cells, which maintained a normal cellular morphology (**Fig. 2**). The results of the measurement of cell area show the significant difference between the control culture of uninfected cells and the cells exposed to *S. aureus* MW2 at MOI 20∶1 for 72 h (**Fig. S2**). Moreover, the effect was not observed when heat-killed *S. aureus* bacteria were used under the same conditions, suggesting that live *S. aureus* are needed to alter the cell cycle (see **Fig. S3**).

### *S. aureus* slowed down proliferation of bovine and human epithelial cells

**Figure 3:**
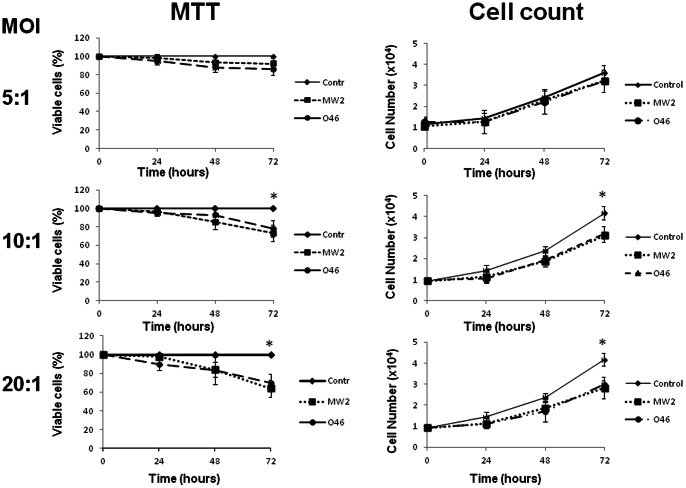
*S. aureus* inhibited cell growth. MAC-T cells were exposed to *S. aureus* MW2 or O46 for 2 h at MOIs ranging from 5∶1 to 20∶1 and further incubated for 24 h, 48 h and 72 h (control: black rhombus; MW2: black square; 046: black circle) and the number of cells was determined (cell count). Cell viability was evaluated by MTT. The results are shown as the percentage of the control. Data are presented as mean +/− SD. Each experiment was done in triplicate. The differences among the groups were assessed by ANOVA. Tukey's Honestly Significant Difference test was applied for comparison of means.

The first results suggested a possible cell cycle alteration induced by *S. aureus* invasion. To understand if host cell proliferation was affected by *S. aureus* invasion, viability and proliferation of the infected cells were estimated by MTT analysis and trypan blue exclusion. The experiments were carried out on cells exposed to *S. aureus* at MOIs ranging from 5∶1 to 20∶1 for 2 h, followed by a gentamicin treatment. The analysis of viability by MTT analysis did not reveal any differences between the MAC-T control cells and the cells exposed to *S. aureus* at MOI 5∶1 (**Fig. 3**) up to 72 h post-infection. Increasing the MOI to 10∶1 resulted in a small but significant (20%) decrease of cells in *S. aureus*-treated cultures, 72 h post-infection. The effect was even more prominent at MOI 20∶1, with a 35% decrease: the quantity of live *S. aureus*-treated cells was 65% that of control cells 72 h post-infection. Both *S. aureus* strains gave similar results on the two cell lines. The proliferation assay was performed by cell counts using a hemacytometer and confirmed the results obtained with MTT (**Fig. 3**). No significant difference was observed between the number of control cells and the number of cells exposed to *S. aureus* at MOI 5∶1 (**Fig. 3**). Increasing the MOI to 10∶1 revealed an inhibition of proliferation at 72 h, resulting in an 18% decrease in the cell count compared to uninfected cells, whereas no significant difference was observed at 24 h and 48 h post-infection. Increasing the MOI to 20∶1 72 h post-infection resulted in a 27% decrease in cell counts.

The MTT assay does not make it possible to distinguish whether or not *S. aureus* exerts a cytostatic or a cytotoxic effect [46]. We therefore investigated the bacterial effect on cell cytotoxicity using trypan blue exclusion. There were no statistically significant differences between *S. aureus*-treated MAC-T cells and control cells at MOIs ranging from 5∶1 to 20∶1 72 h post-infection for the two strains (**Fig. S4**). Similar results were obtained when HeLa cells were treated with the *S. aureus* (data not shown). Altogether, these results demonstrated that the growth of MAC-T and HeLa cells was slowed down following exposure to *S. aureus* in a dose- and time-dependent fashion.

### Exposure of epithelial cells to *S. aureus* dramatically decreased the number of mitoses

The effect of *S. aureus* invasion on epithelial cells was further investigated by estimating the mitotic index in DAPI-stained cells. Synchronized by DTB, HeLa or MAC-T cells were infected either by human MW2 or by ruminant O46 strains, and the mitotic indexes in infected cells were compared to those in uninfected cells 25 h post-infection. Asynchronous uninfected cells were used as a control.

**Figure 4:**
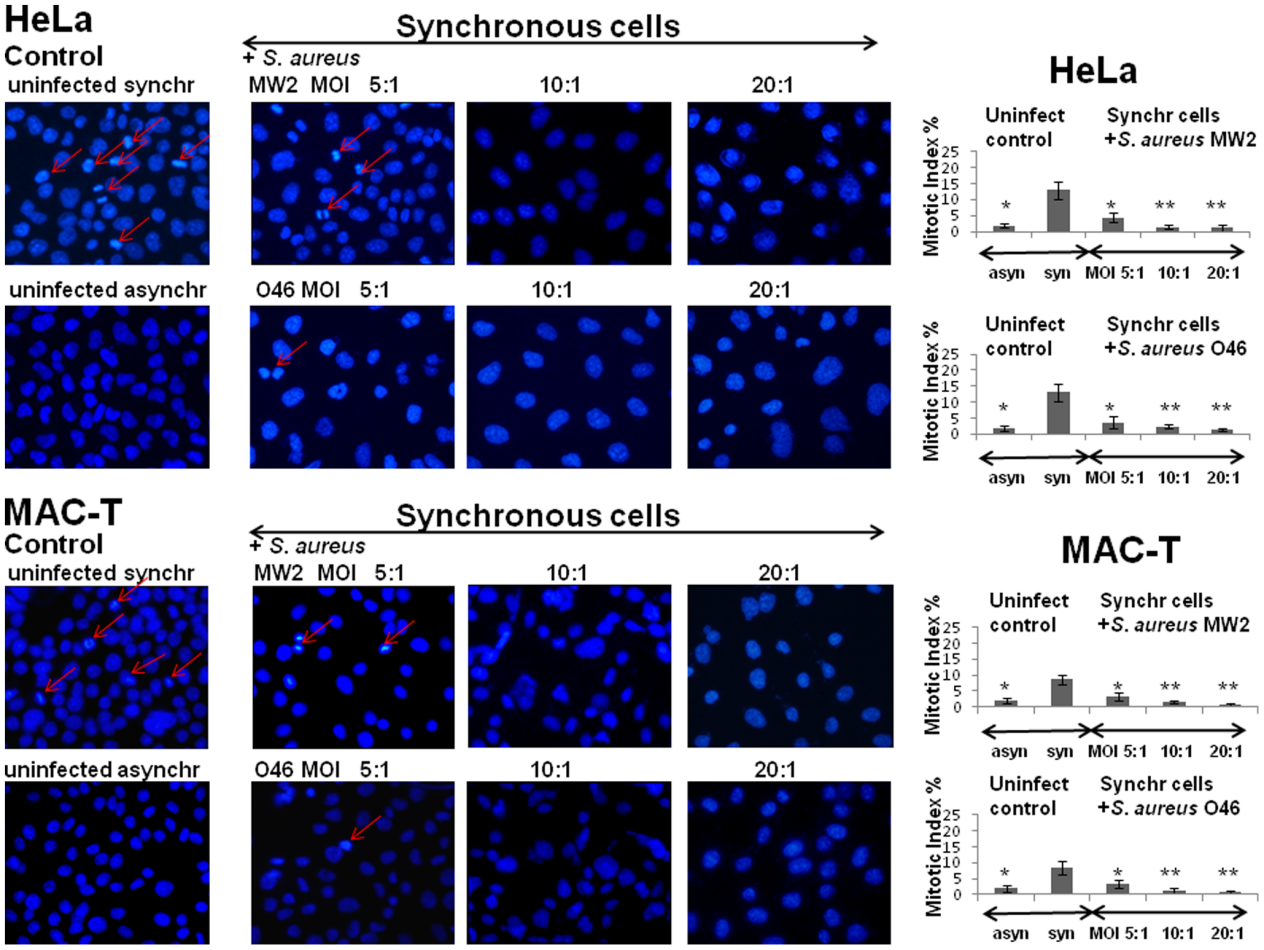
Substantial drop in the mitotic index in *S. aureus*-infected cells. HeLa or MAC-T cells were synchronized by DTB and were then exposed to MW2 or O46 strains at MOIs ranging from 5∶1 to 20∶1 for 2 h, followed by incubation in cDMEM-Gent100 for 2 h, and further incubated for 25 h. After centrifugation, the cells were fixed and stained with DAPI. Red arrows indicate the mitotic cells. The mitotic indexes in infected and in uninfected synchronous cells were evaluated by microscopic observation using ×400 magnification. Data are presented as mean +/− SD. The differences among the groups were assessed by ANOVA. (*) P-values <0.05 and (**) P-values <0.01 compared with the control were considered to be significant. Tukey's Honestly Significant Difference test was applied for comparison of means between the groups.

The mitotic index in the synchronous uninfected cells was higher and was estimated as 13%±2.6%, compared to asynchronous uninfected HeLa cells (2.3%±1.8%) (**Fig. 4**). Exposure of synchronous cells to *S. aureus* led to a statistically significant dose-dependent decrease in the number of cells in the mitotic phase. In synchronous HeLa cells, the mitotic index dropped down to 4.5%±1% in cultures exposed to *S. aureus* MW2 at MOI 5∶1 ((*) p<0.05 vs. uninfected cells), to 2.4%±0.7% at MOI 10∶1, and to 1.1%±0.5% at MOI 20∶1 ((**) p<0,01 vs. uninfected cells) at the same time point. An analogous decrease in the mitotic index was observed in synchronized HeLa cells exposed to the ruminant O46 strain. Similar results were also obtained with MAC-T cells exposed either to human MW2 or to ruminant O46 strains (**Fig. 4** **)**.

Exposure of the HeLa or MAC-T cells to another well-characterized animal strain, O11, which was shown to induce gangrenous mastitis in a ewe model [26], [27], resulted in an equivalent decrease of the mitotic index (**Fig. S5).** The mitotic index dropped in synchronized uninfected HeLa cells from 13%±2.6%, down to 4.1%±1.1% in cultures exposed to ruminant O11 at MOI 5∶1 ((*) p<0.05 vs. uninfected cells), to 1.4%±0.9% at MOI 10∶1, and to 1.1%±0.7% at MOI 20∶1 ((**) p<0,01 vs. uninfected cells) at the same time point. Analogous results were also obtained with MAC-T cells exposed to ruminant O11 strain. The mitotic index in the synchronous uninfected MAC-T cells was estimated as 8.9%±3%, compared to asynchronous uninfected cells (2%±1.1%). Exposure of synchronized MAC-T cells to ruminant O11 strain resulted in a statistically significant dose-dependent decrease in the number of cells in the mitotic phase: 3.3%±1% at MOI 5∶1, 1.3%±0.8% at MOI 10∶1 and 0.8%±0.5% at MOI 20∶1 (**Fig. S5**).

### *S. aureus* induced a G2/M-phase transition delay in epithelial cells

**Figure 5:**
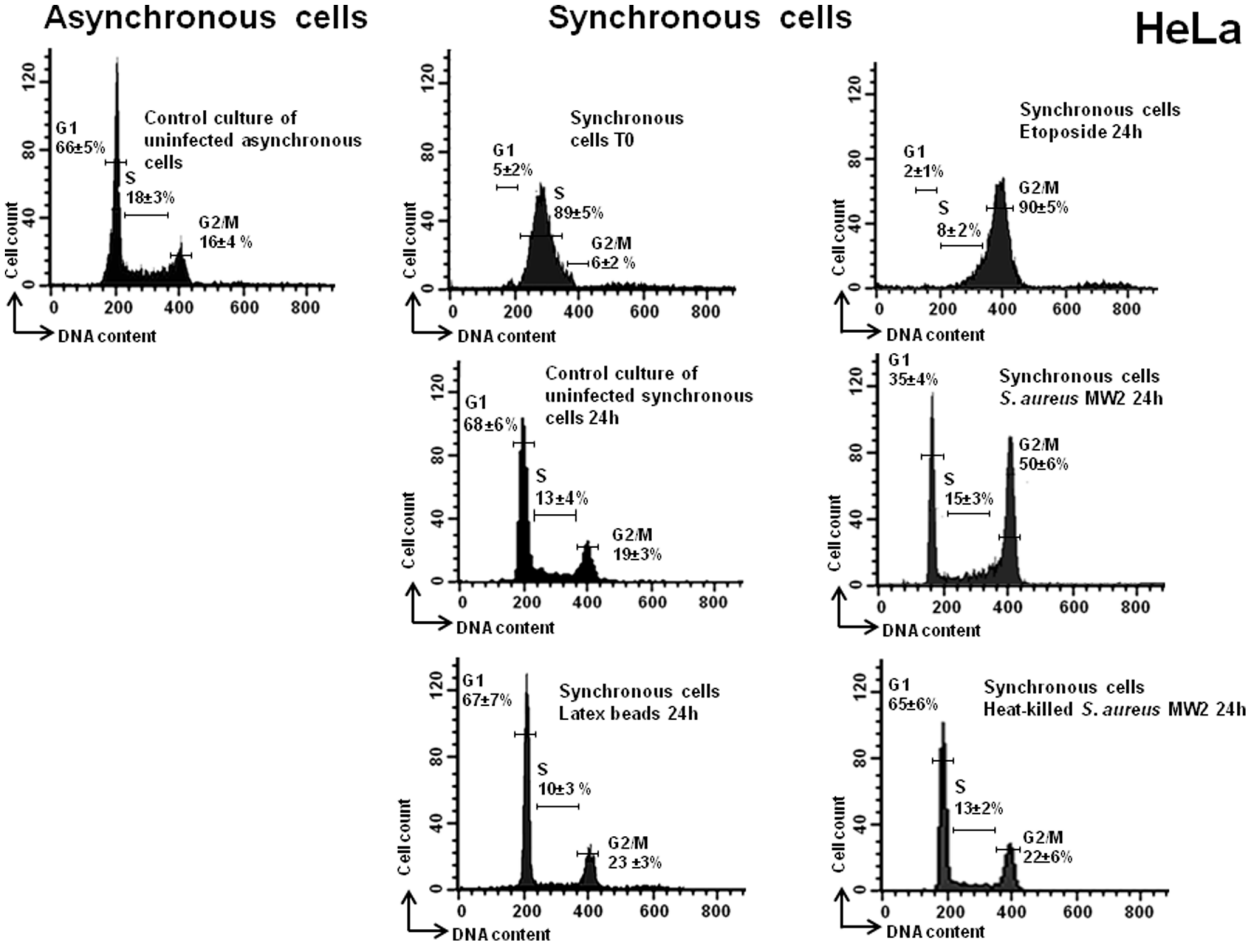
*S. aureus* induced a G2/M phase transition delay. HeLa cells were synchronized by DTB and were exposed to live or heat-killed bacteria (MW2) at MOI 20∶1, to the latex beads or to the media alone for 2 h, followed by incubation in cDMEM-Gent100 for 2 h, and further incubated for 24 h. Some cells were exposed to etoposide for the same length of time as the exposure to bacteria. Detached cells were then combined with adherent cells and stained with PI. Cell cycle phases of PI-stained cells were monitored by FACS. The data were collected from 20,000 cells and analysis was performed with Cell Quest software. The average percentage of cell cycle phase ± SD is indicated. Each experiment was performed at least five times. Exposure of the cells to live *S. aureus* bacteria induced an increase in the number of cells in the G2/M phase, (*) p<0.05.

The inhibition of proliferation, the decrease of the mitotic index and the enlargement of the infected cells could be related to a host cell cycle alteration during the infection. In order to investigate whether or not the exposure of epithelial cells to *S. aureus* results in host cell cycle alteration, HeLa cells, 4 h after DTB synchronization (i.e., middle of the S phase of the cell cycle), were exposed either to live or heat-killed bacteria, to the medium alone, or to latex beads for 2 h, followed by 2 h of incubation in cDMEM-Gent100 and a subsequent 24 h of incubation in cDMEM-Gent25. DNA analysis of synchronized cells (4 h after the second block release) confirmed that the majority of the cells were in the S phase (up to 89% of the cells), as shown in **Fig. 5**. It is worth noting that this is in agreement with the recognized evaluation of the phases of the HeLa cell cycle (approximately 10 h, 8 h, 3 h and 1 h, for G1, S, G2 and M, respectively) [47]. Some cells were exposed to etoposide for the same length of time that they were exposed to bacteria. Analysis of etoposide-treated cells revealed that the majority of the cells had a 4n DNA content, corresponding to cells in the G2 phase (90%, **Fig. 5**), since etoposide induces arrest of the cell cycle at the G2 phase [48]. Analysis of DNA content 24 h after elimination of the extracellular bacteria revealed a significant increase in the number of cells in the G2/M phase, compared to the synchronous uninfected ones. Experiments showed an increase in the proportion of cells in the G2/M phase from 19±3% in uninfected cells and up to 50±6% in infected cells (**Fig. 5**, MOI 20∶1; representative result) (p<0.05). Exposure to 2-µm latex beads did not lead to any cell cycle phase alteration. These results demonstrate that *S. aureus* induces a G2/M-phase transition delay in the host cell cycle and support the conclusion that this delay is specific and not caused by the cellular stress related to the presence of extraneous particles. To understand whether live bacteria are required to induce G2/M-phase transition delay, heat-killed *S. aureus* cells at the same concentrations were used. The G2/M-phase transition delay was not observed when heat-killed *S. aureus* bacteria were used, i.e., there were no significant differences in the cell cycle distribution of synchronized HeLa cells exposed or not to the heat-killed bacteria at any of the time intervals tested (**Figs. 5** **, S6**).

### Cells exposed to *S. aureus* during the S phase are transiently delayed at the subsequent G2/M phase

**Figure 6:**
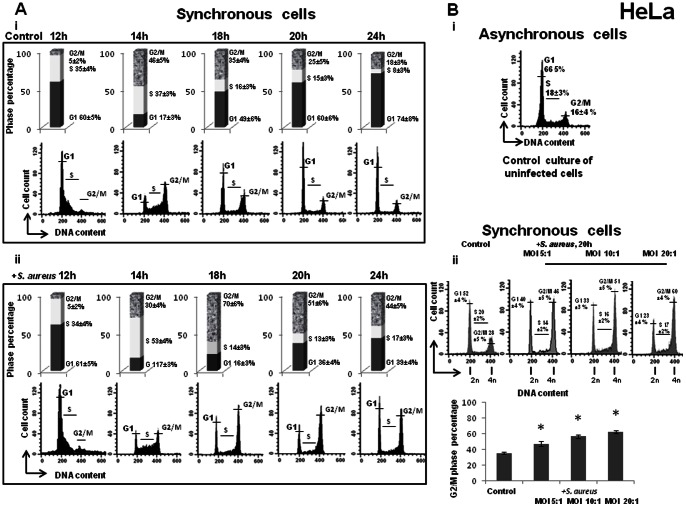
*S. aureus* induced dose-dependent G2/M phase transition delay in the subsequent G2/M phase. **A.** HeLa cells were synchronized by DTB and were exposed to *S. aureus* bacteria (MW2) at MOI 20∶1 or to the media alone for 2 h, followed by incubation in cDMEM-Gent100 for 2 h, and subsequently incubated for an additional 12 h, 14 h, 18 h, 20 h and 24 h. Detached cells were then combined with adherent cells and stained with PI. Cell cycle phases of PI-stained cells were monitored by FACS. The data were collected from 20,000 cells and analysis was performed with Cell Quest software. Each experiment was performed four times. The number of cells in different phases is presented on the histograms. The values shown are those of a representative assay out of the four assays performed. Exposure of the cells to *S. aureus* bacteria induced a G2/M phase transition delay in a time-dependent manner. **B**. HeLa cells were synchronized by DTB and were exposed to *S. aureus* bacteria (MW2) at different MOIs ranging from 5∶1 to 20∶1, followed by incubation in cDMEM-Gent100 for 2 h, and further incubated for an additional 20 h. Detached cells were then combined with adherent cells and stained with PI. Cell cycle phases of PI-stained cells were monitored by FACS. The data were collected from 20,000 cells and analysis was performed with Cell Quest software. The percentage of cells is presented on the graph as a function of bacterial concentration. The data correspond to a representative experiment out of the three assays performed and are presented as mean +/− SD. Tukey's Honestly Significant Difference test was applied for comparison of means between the groups. (*) P-values <0.05 compared with control were considered to be significant. Exposure of the cells to *S. aureus* bacteria induced a G2/M phase transition delay in a dose-dependent manner.

Previous experiments revealed an increase in the proportion of the G2/M phases of *S. aureus*-infected cells. However, the cells exposed in the S phase could be delayed during the same cycle or during the following one since cells are likely to arrive at a new G2 phase after a 24 h period. To test this hypothesis, we investigated the distribution of cell cycle phases at different periods post-infection. Cells in late S phase (6 h after DTB release) were exposed to *S. aureus* for 2 h, followed by incubation in cDMEM-Gent100 for 2 h, which eliminates the extracellular bacteria and retains the intracellular bacteria. After the additional periods of incubation (12 h, 14 h, 18 h, 20 h and 24 h), the cell DNA content was then examined by FACS. The mean values of one representative result from three independent experiments are illustrated in **Fig. 6A**. The cell cycle distribution of the control asynchronous cells is shown in **Fig. 6B** **i**. Analysis of DNA content of synchronous cells showed no difference between control and infected cells 12 h after elimination of extracellular bacteria (MOI 20∶1). After a 14 h interval, a large majority of cells had initiated a new cycle. Following 18 h of incubation, cells no longer exited from the G2/M phase. The number of cells in the G2/M phase was almost twice as high in *S. aureus*-treated cells as in non-infected ones: 70%±4% vs. 35%±3%. Two hours later (20 h of incubation), the number of cells in the G2/M phase was 51%±3% vs. 20%±3% of cells in the G1 phase. After 24 h of incubation, the mean proportion of cells remaining in the G2/M phase was still higher in infected cells (44%±3%) than in non-infected cells (18%±3%). These results showed that most of the cells had initiated a new cycle, completed the S phase and were delayed at the subsequent G2/M phase. The effect was dose-dependent: an increase of MOI from 5∶1 to 20∶1 resulted in an increase in the number of cells in the G2/M phase (**Fig. 6B**). Altogether, these results suggested that *S. aureus*-infected cells have to pass through the S phase in order to trigger G2/M-phase transition delay.

### Exposure of cells to *S. aureus* results in the accumulation of phosphorylated Cdk1 and unphosphorylated histone H3

**Figure 7:**
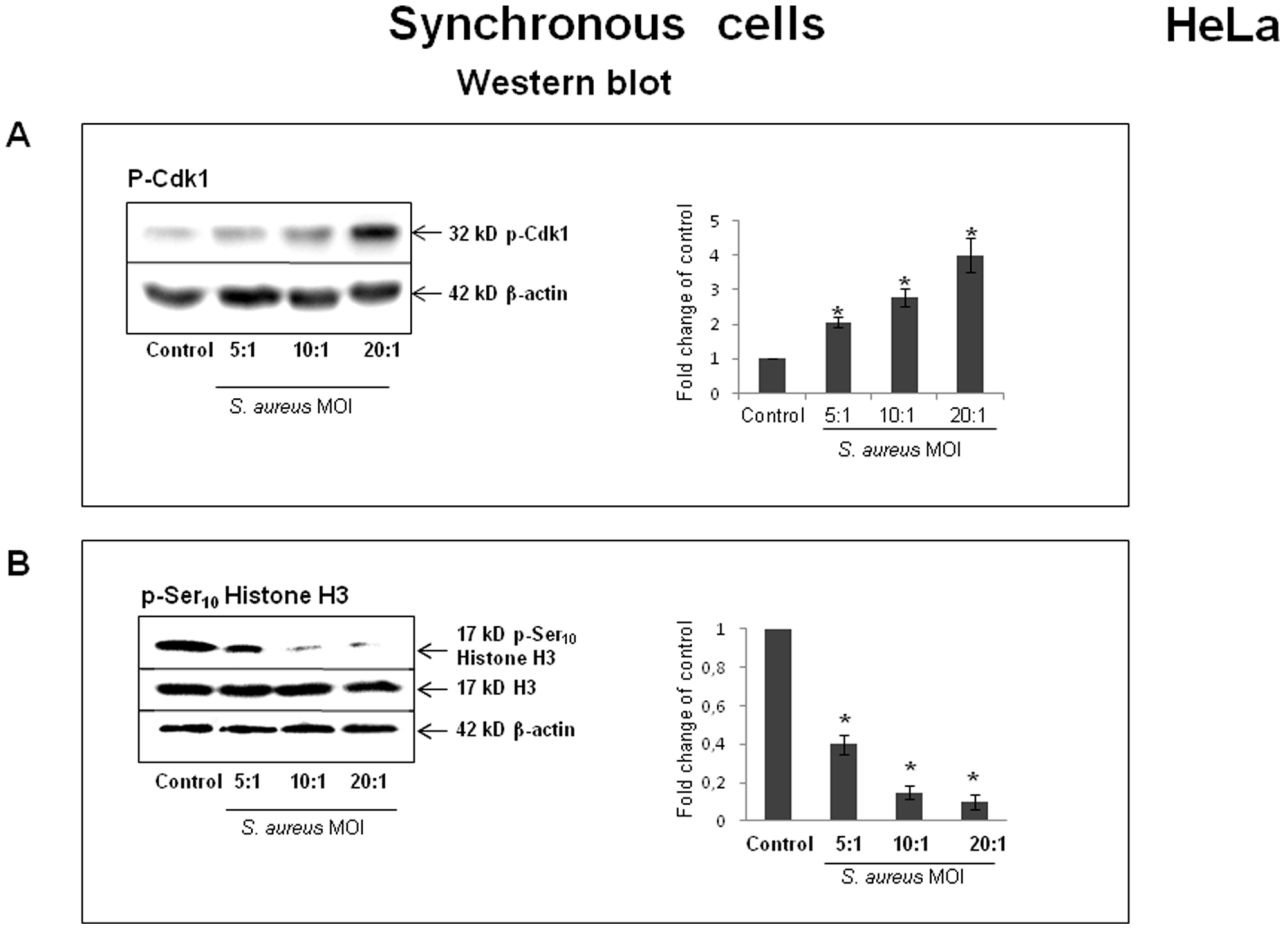
*S. aureus*-induced accumulation of phosphorylated Cdk1 and unphosphorylated Ser10 Histone H3. HeLa cells were synchronized by DTB and were exposed to *S. aureus* bacteria (MW2) at MOIs ranging from 5∶1 to 20∶1 for 2 h, followed by incubation in cDMEM-Gent100 for 2 h, and further incubated for 20 h. Cells were then suspended in Laemmli loading buffer, and Western blot analysis, either with anti-phospho-Cdk1 antibodies or anti-p-Ser10 Histone H3 antibody, was performed as described in the Materials and Methods section. The chemiluminescence reaction was visualized and processed with a G:BOX imaging system. Blots are representative of three separate experiments. Data are presented as mean ± SD from three densitometry scans. Tukey's Honestly Significant Difference test was applied for comparison of means between the groups. (*) P-values <0.05 compared with the control were considered to be significant.

Flow cytometry data clearly showed an accumulation of *S. aureus*-infected cells in the G2/M phase. It was therefore important to determine whether *S. aureus*-infected cells were delayed in the G2 phase and failed to enter mitosis or, instead, were blocked in the M phase and failed to complete mitosis. Cycline-dependent kinase Cdk1 is one of the key players of the G2-M transition [49]. Dephosphorylation of the Thr14 and Tyr15 residues of Cdk1 at the end of the G2 phase is essential for triggering mitotic entry. In order to analyze *S. aureus*-induced G2 delay, the phosphorylated status of Cdk1 was assessed by Western blot analysis using anti-phospho-Cdk1 antibody 20 h after elimination of the extracellular bacteria. The protein load was normalized with anti-actin antibodies. All of the synchronized HeLa cell cultures used for semi-quantitative Western blotting were simultaneously analyzed by FACS for cell cycle determination. The relative levels of phosphorylated (Tyr15) Cdk1 (inactive form) increased in a dose-dependent fashion in the cells exposed to *S. aureus* (**Fig. 7A**), indicating that most of the cells were delayed in the G2 phase and were prevented from entering mitosis rather than being arrested in the M phase. The effect of *S. aureus* infection on the initiation of mitosis was also examined by determining the abundance of the established mitosis marker, histone H3. Phosphorylation of histone H3 at Ser10 is indeed associated with the condensation of chromosomes during mitosis. The addition of *S. aureus* considerably decreased the relative level of phosporylated histone H3 (p-Ser10 Histone H3) in a dose-dependent manner (*P<0.05), while the total level of histone H3 remained constant in all of the cell samples (**Fig. 7B**). We next examined whether this *S. aureus-*induced decrease of p-Ser_10_ Histone H3 level was associated with the observed reduction of the mitotic cell number after infection. During mitosis, histone H3 phosphorylation at Ser10 starts in prophase, with the maximal level in metaphase, followed by a decrease of phosphorylation during the progression to telophase. The time of analysis was chosen in order to detect the mitotic cells.

**Figure 8:**
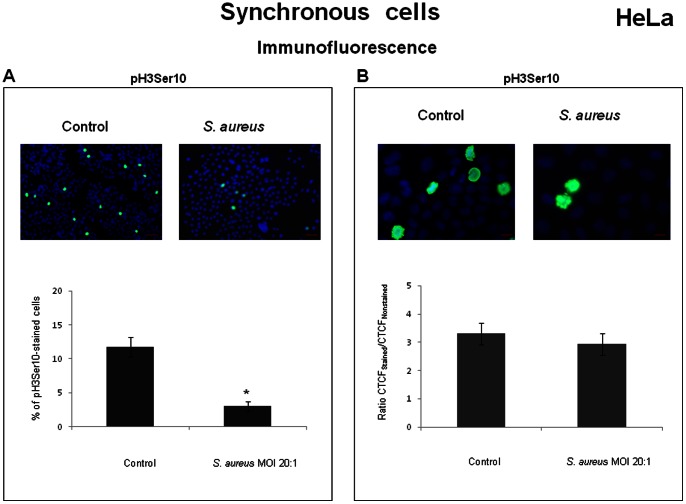
*S. aureus* decreased the number of p-Ser10 Histone H3-positive nuclei in infected culture. HeLa cells were synchronized by DTB and were then exposed to MW2 strain at MOI 20∶1 for 2 h, followed by incubation in cDMEM-Gent100 for 2 h, and further incubated for 20 h. After centrifugation, the cells were fixed, stained with anti-p-Ser10 Histone H3 antibody and DAPI, and observed under the microscope. The relative level of fluorescence (CTCF) of p-Ser10 Histone H3-positive mitotic nuclei was measured with ImageJ software. Data are presented as mean +/− SD. Tukey's Honestly Significant Difference test was applied for comparison of means between the groups. (*) *P<*0.05 compared with the synchronous control. A decrease in the number of p-Ser10 Histone H3-positive nuclei was observed in *S. aureus-*infected culture (A, scale bars: 100 µm). Estimation of the relative fluorescence intensity of p-Ser10 Histone H3-stained mitotic cells did not reveal statistically significant differences between infected and non-infected cultures (B, scale bars: 10 µm). The values are from the three assays performed.

A strong decrease in the number of p-Ser10 Histone H3 positive nuclei was observed in *S. aureus-*infected culture (**Fig. 8A**). However, the estimation of the relative fluorescence intensity of individual mitotic cells (metaphase) stained with the anti-p-Ser10 Histone H3 antibody did not reveal a statistically significant difference between infected and uninfected cultures (**Fig. 8B**). Thus, the dramatic decrease of p-Ser10 Histone H3 level detected by Western blotting was mostly due to a strong decrease in the number of p-Ser10 Histone H3- positive nuclei after *S. aureus* infection. Altogether, these results suggest that most of the infected cells failed to enter mitosis and accumulated in the G2 phase.

### Delay of the HeLa cells in the G2 phase results in an increased infective efficiency of *S. aureus*

It was shown for some pathogens that cause host cell cycle alterations that the stage where a cell cycle delay occurs is advantageous for the pathogen's proliferation [41], [49]. We investigated whether *S. aureus* G2/M-induced transition delay of the host cells had an effect on bacterial proliferation. To address this issue, we used inhibitors of cell cycle progression, which were reported to arrest the eukaryotic cells in the G1 or G2 phases. The inhibitors alone had no effect on *in vitro* growth of *S. aureus* bacteria since the growth curves of bacteria in DMEM in the absence of eukaryotic cells with or without inhibitors were similar, as was estimated spectrophotometrically and confirmed by plate counts. The optical density after 24 hours of growth reached 0.6 at 600 nm, corresponding to approximately 10^8^ CFU/ml for all bacterial samples. HeLa cells were pre-incubated for 3 h before infection and then throughout infection until analysis in the inhibitor-containing medium to arrest the cells in the G_1_ or G_2_ phase, as described in the Materials and Methods section. The CFU value and HeLa cell cycle at 0 h, 2 h, 4 h and 20 h from the beginning of incubation in cDMEM-Gent100 were determined. There were no significant differences in the number of CFUs between the cells treated with inhibitors and those without inhibitors 2 h post-infection (T0, the beginning of the gentamicin treatment) (data not shown). At that time, the CFU value corresponds to both adherent and internalized bacteria. Analysis of the cell cycle distribution at T0 did not reveal any significant difference between the cells treated or not with the inhibitors. The cell cycle distribution was similar in treated and untreated cells: 60%, 19% and 21% for the G1, S and G2/M phases, respectively.

**Figure 9:**
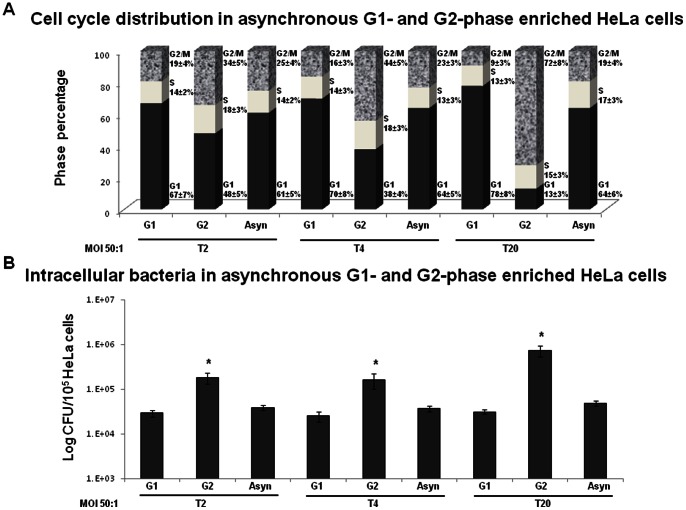
Blocking HeLa cells in the G2 phase results in an increased internalization and in an increased intracellular replication of *S. aureus.*. HeLa cells were treated with either 10 µM CDK4/6 inhibitor IV (enrichment in the G1 phase) or 9 µM of RO-3306 (enrichment in the G2 phase) 3 h before the addition of *S. aureus* at MOI 10∶1 and 50∶1 for 2 h (T0). The cells then were incubated in cDMEM-Gent100 for an additional 2 h, 4 h (T4) or 20 h (T20). The proportions of cells in the different cell cycle stages at T2, T4 and T20 were determined by FACS (A). The CFU of intracellular *S. aureus* at T2, T4 and T20 was determined by plate count. CFU values were normalized to 10^5^ HeLa cells (B). Experiments were performed in triplicate. The data are presented as mean +/− SD. Tukey's Honestly Significant Difference test was applied for comparison of means between the groups. (*) *P<*0.05 compared to asynchronously grown cells.

Incubation of the infected cells for a subsequent 2 h in the inhibitor/gentamicin-containing medium (T2) resulted in cell enrichment either in the G1 phase or in the G2 phase. The CFU number (corresponding here to the internalized bacteria) normalized to 10^5^ HeLa cells in the G2 phase-enriched cells, infected at MOI 50∶1, was approximately 6-fold higher than that of the cells enriched in the G1 phase or that of the asynchronous cells (no inhibitor treatment) (P<0.05) (**Fig. 9B**). Approximately 1±0.3% of inoculum was internalized by asynchronous or G1-phase enriched cells, whereas 5.9±0.8% of inoculum was internalized by G2-enriched cells. These results suggest that the G2 phase of the host cells was advantageous for staphylococcal internalization. In order to verify if intracellular replication occurs, the CFU 4 h and 20 h after the beginning of gentamicin treatment was evaluated. Incubation of the infected cells for 4 h in the inhibitor/gentamicin-containing medium (T4) resulted in cell enrichment either in the G1 phase or in the G2 phase (**Fig. 9B**). There was no significant difference between the number of intracellular bacteria 2 h (T2) and 4 h (T4) after the beginning of gentamicin treatment.

Incubation of the infected cells for 20 h in the inhibitor/gentamicin-containing medium resulted in accumulation of the cells either in the G1 phase or the G2 phase (**Fig. 9A**). The CFU number in the G1 phase-enriched and asynchronous cells at T20 was 1.2-fold and 1.4-fold higher than the value at T2. These results indicate the bacterial replication 20 h post-infection. An even greater difference was observed for the CFU value in the G2 phase-enriched cells. The CFU value in the G2 phase-enriched cells (corresponding to intracellular bacteria) normalized to 10^5^ HeLa cells at T20 was 3.7-fold higher than the value at T2 (**Fig. 9B**). Similar results were observed for MOI 10∶1 (data not shown). These data suggest that the G2 phase is preferential for the intracellular proliferation of internalized *S. aureus*. Therefore, the G2 phase is advantageous for staphylococcal infective efficiency, which depends on *S. aureus* internalization and replication.

## Discussion

Maintenance of an intact epithelial barrier constitutes an important defense mechanism against infections. The integrity of this barrier is also dependent on a continuous renewal of epithelial cells fueled by proliferation of progenitor cells [50]. Many bacteria have developed specialized strategies to disrupt key eukaryotic cell functions in order to establish persistent colonization, some of which rely on cell cycle alteration [32]. Fidelity of the cell cycle is maintained by DNA damage checkpoint mechanisms that validate the integrity/accuracy of DNA replication. Activation of these checkpoints results in cell cycle arrest so that DNA can be repaired or, in the case of severe damage, progress to programmed cell death [51]. The current experiments were designed to investigate the effect of a staphylococcal infection on epithelial cells and to examine its ability to affect the host cell cycle. Exposure to live *S. aureus* strains of human or animal origin resulted in the induction of a cytopathic effect in the epithelial cells, characterized by a progressive enlargement of the whole body (including the area of the cytoplasm) of infected cells 72 h post-infection. Cell cycle delay/arrest (following DNA damage, for example) is usually associated with cell cytoplasm enlargement, which is a senescence-related phenotype [52]. The cytopathic effect, resulting in morphological changes of the infected cells such as cell enlargement, is a recognized feature of certain cyclomodulins. For example, this cytopathic effect was observed upon (i) infection of different mammalian cells (including HeLa cells) with pathogenic *E. coli* strains [31], [32]; (ii) cell intoxication with the cytolethal distending toxin CDT [53]; and (iii) infection of HeLa cells with Cif-producing bacteria [13]. Live bacteria were required to observe this cytopathic effect. Exposure to heat-killed *S. aureus* bacteria did not lead to any cytopathic effect.

Our experiments showed that the proliferation rate of the epithelial cells was slowed down in *S. aureus*-infected cultures. These observations are consistent with other findings that reveal the inhibition of MAC-T cell proliferation by *S. aureus* culture supernatants [54], and with transcriptome analysis, which showed alterations in the expression profiles of genes involved in cell proliferation in *S. aureus*-infected cells or tissues [21], [22].

Analysis of cell cycle-synchronized cultures revealed a drastic decrease of the mitotic index in infected cells compared to uninfected ones at the same time points. Similar cytopathic effects, inhibition of cell proliferation and decrease of the mitotic index were observed on both human and bovine cells with the two *S. aureus* strains tested, regardless of their human or animal origin, suggesting that this effect does not depend on host-specific relationships.

The inhibition of proliferation in addition to the drop in the mitotic index and enlargement of the infected cells pointed out the possibility of host cell cycle alteration during infection. Cell cycle distribution determined by flow cytometry with synchronized cells revealed the *S. aureus*-induced G2/M phase transition delay and showed that the degree of cell cycle delay was correlated with the multiplicity of the infection, demonstrating a dose-dependent effect on the cell cycle control mechanisms. Pathogen-induced cell cycle delay is likely to intervene through various transduction pathways that require a fine-tuning of growth-stimulating and growth-inhibiting processes.

Transcriptome analyses of human corneal epithelial cells exposed to *S. aureus* and *S. aureus*-infected bovine mammary tissue reveal an alteration in the expression profiles of genes that affect the immune response and cell cycle control [21], [22]. The *S. aureus*-induced G2 phase delay demonstrated here suggests that the complex network of bacteria-host cell interactions affects the expression of genes related to the regulation of cell cycle transition. This delay was specific and not caused by the cellular stress related to the presence of the extraneous matter, as was shown in the experiments with the latex beads. Live bacteria were indispensable for the induction of the observed G2 phase delay since heat-killed *S. aureus* did not induce any delay. The way in which the G2 phase delay demonstrated here fits into the multifaceted system of bacteria-host cell relationships that affect the expression of genes involved in cell cycle regulation remains to be elucidated.

Most cells exposed to *S. aureus* late in the S phase are not delayed at the current cycle but at the subsequent one. This finding implies that passage through the S phase is necessary for the triggering of the G2 delay. It can be hypothesized here that *S. aureus* interferes with a transduction pathway that begins in the S phase during DNA replication and that may be related to a DNA damage checkpoint [55]. Therefore, *S. aureus* may either induce DNA damage or interfere with components of the DNA damage checkpoint system. For many cell lines, Cdk1 phosphorylation is involved in DNA-damage checkpoint response [56]. Dephosphorylation of Cdk1 on Tyr-15 and Tyr-14 residues is necessary for its activation and for the entry into mitosis [57]. Our experiments showed that the G2 phase delay was correlated with a dose-dependent accumulation of the non-active phosporylated form of Cdk1, suggesting that most of the cells were prevented from entering mitosis. Inhibition of mitosis entry was further confirmed by observation of the accumulation of unphosphorylated histone H3 (a marker of mitosis), which was correlated with a reduction of the mitotic cell number in *S. aureus*-treated cells. Further study is necessary to determine whether *S. aureus* acts directly on the dephosphorylation machinery or reacts through a cyclin-dependent kinase inhibitor.

The question then arises as to the biological significance and the advantage for *S. aureus* to induce G2 phase delay. It was shown that respiratory syncytial virus induces G1/G0 phase arrest of the airway cells and that this phase was favorable for virus production [41].

Although not generally considered an intracellular pathogen, *S. aureus* can be internalized by host cells [8], [43]. The results of the internalization assay that we performed show that *S. aureus* was internalized inside of the human epithelial HeLa cells and that the percentage of internalized bacteria in asynchronous cells was similar to the one described by Kintarak et al. [58]. The internalization rate of *S. aureus* was higher for the HeLa cells in the G2 phase of the cell cycle compared to the asynchronous cells, suggesting the advantage of induced G2 phase delay in bacterial invasion.

Since intracellular replication of *S. aureus* inside epithelial cells within a 24 h period has already been reported [8], it is plausible that the induction of a host cell cycle alteration by *S. aureus* leads to improved intracellular bacterial survival or increased proliferation. The analysis of staphylococcal proliferation in asynchronous cells and in cell cultures enriched in the G1 or G2 phase showed here that the G2 phase is advantageous for bacterial intracellular replication and may contribute to the persistence of the infection. Whether it is due to *S. aureus* activity on the cell proliferation machinery or to a reaction of the host cells against the staphylococcal infection is still unknown. Further research is required to fully understand the complex mechanisms of the cell cycle alteration induced by *S. aureus*.

Our findings open new perspectives for the investigation of the physiopathology of *S. aureus*-induced human and animal diseases at the molecular level and for the development of innovative approaches in the treatment of staphylococcal infections that target host cell cycle regulation.

## Conclusion

*S. aureus* invasion induced a cytopathic effect, resulting in the enlargement of host epithelial cells and in the decrease of the mitotic index of the infected cells. Epithelial cells infected by *S. aureus* proliferate more slowly than uninfected cells and accumulate in the G2 phase of the cell cycle. Live bacteria were indispensable for the induction of the G2/M phase transition delay. The observed G2/M transition phase delay was associated with the accumulation of inactive Cdk1 and unphosphorylated histone H3, which was correlated with a reduction of the mitotic cell number. Additionally, we showed that the G2 phase was preferential for bacterial internalization and intracellular replication. Overall, the results suggested that the G2 phase delay of the infected epithelial cells may be one of the mechanisms used by *S. aureus* to survive and to propagate inside the host.

## Supporting Information

Figure S1 **The visualization of** ***S. aureus*** **bacteria in the infected MAC-T cells.** MAC-T cells were grown on cover slips and were then exposed to SYTO 9-stained MW2 strain for 2 h, followed by incubation in cDMEM-Gent100 for 2 h. The cells then were fixed, stained with DAPI and observed under the microscope. Red arrows indicate *S. aureus* bacteria.
(TIF)

Figure S2 **Evaluation of HeLa and MAC-T cell size.** HeLa or MAC-T cells were grown on cover slips and were then exposed to MW2 strain at MOI 20∶1 for 2 h, followed by incubation in cDMEM-Gent100 for 2 h. The cells then were further incubated in cDMEM-Gent25 for 72 h. The cells were then fixed and stained with DAPI. The merged images of phase contrast and DAPI-stained cells were observed under the microscope. The measurement of the area of the whole cells was performed with ImageJ software. Data are presented as mean (arbitrary unit of the area) +/− SD. Three independent assays were performed. Tukey's Honestly Significant Difference test was applied for comparison of means between the groups.
(TIF)

Figure S3 **Exposure of eucaryotic cells to heat-killed** ***S. aureus*** **bacteria do not induce a cytopathic effect.** Human HeLa or bovine MAC-T cells were exposed for 2 h to live or heat-killed *S. aureus* MW2 at MOI 20∶1 and further incubated for 72 h. The cells then were fixed, stained with DAPI, and observed using ×400 magnification. The merged image of phase contrast and DAPI-stained cells is presented. Red arrows indicate the enlarged cells in infected cell cultures. Microscopic observation revealed the enlargement of the cells exposed to live *S. aureus* bacteria. One representative experiment out of the three is shown. Scale bars: 10 µm.
(TIF)

Figure S4 **Trypan blue dye exclusion assay.** MAC-T cells were exposed to *S. aureus* MW2 or O46 for 2 h at MOIs ranging from 5∶1 to 20∶1 and were further incubated for 24 h, 48 h and 72 h (control: black rhombus; MW2: black square; 046: black circle). Cell viability was evaluated by trypan blue exclusion assays. The results were calculated as the percentage of live cells out of the total number of cells. Data are presented as mean +/− SD. The plotted points represent means of at least three independent experiments. Tukey's Honestly Significant Difference test was applied for comparison of means between the groups.
(TIF)

Figure S5 **Decrease of the mitotic index in eucaryotic cells exposed to the O11** ***S. aureus*** **strain.** HeLa or MAC-T cells were synchronized by DTB and were then exposed to O11 *S. aureus* strains at MOIs ranging from 5∶1 to 20∶1 for 2 h, followed by incubation in cDMEM-Gent100 for 2 h, and then further incubated for 25 h. After centrifugation, the cells were fixed and stained with DAPI. Red arrows indicate the mitotic cells. The mitotic indexes in infected and in non-infected synchronous cells were evaluated by microscopic observation using ×400 magnification. Data are presented as mean +/− SD. The differences among the groups were assessed by ANOVA. (*) P-values <0.05 and (**) P-values <0.01 compared with control were considered to be significant. Tukey's Honestly Significant Difference test was applied for comparison of means between the groups.
(TIF)

Figure S6 **G2/M transition delay is induced by live** ***S. aureus*** **bacteria.** HeLa cells were synchronized by DTB and were exposed to live or heat-killed *S. aureus* bacteria (MW2) at MOI 20∶1 for 2 h, followed by incubation in cDMEM-Gent100 for 2 h, and subsequent incubation for an additional 12 h, 14 h, 18 h, 20 h and 24 h. Detached cells were then combined with adherent cells and stained with PI. Cell cycle phases of PI-stained cells were monitored by FACS. The data were collected from 20,000 cells and analysis was performed with Cell Quest software. The number of cells in different phases is presented on the histograms. The values shown are those of a representative assay out of the four assays performed.
(TIF)
