# Supplementary figures and images for: Staphylococcus aureus-Induced G2/M Phase Transition Delay in Host Epithelial Cells Increases Bacterial Infective Efficiency
Source: PLoS One. 2013 May 23;8(5):e63279. doi: 10.1371/journal.pone.0063279 (PMC3662696; doi:10.1371/journal.pone.0063279)

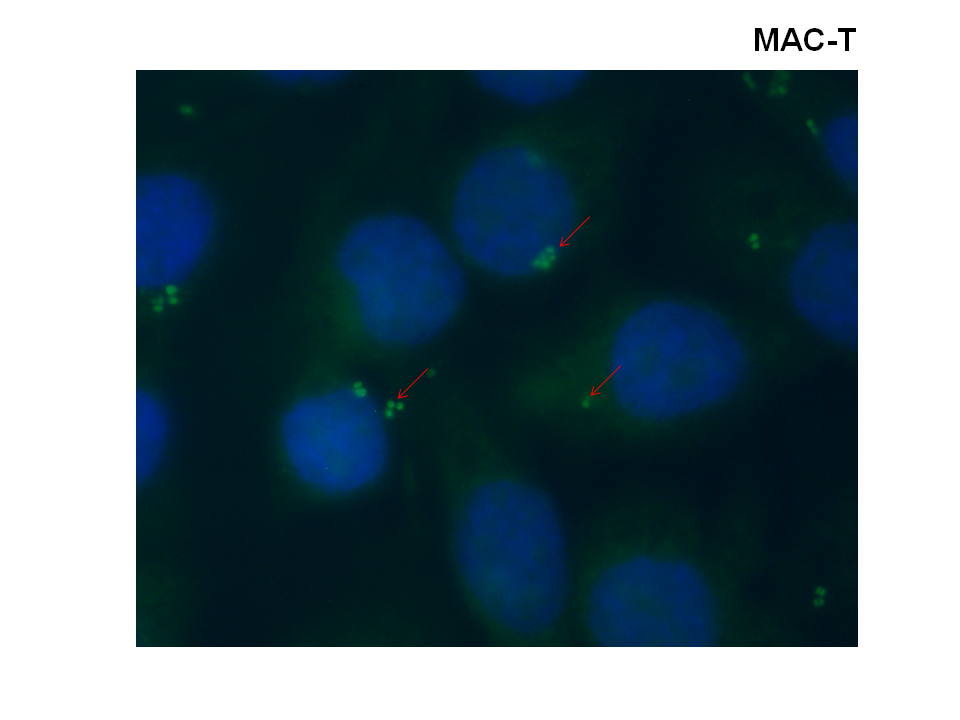

Supplement: Figure S1 — The visualization of S. aureus bacteria in the infected MAC-T cells. MAC-T cells were grown on cover slips and were then exposed to SYTO 9-stained MW2 strain for 2 h, followed by incubation in cDMEM-Gent100 for 2 h. The cells then were fixed, stained with DAPI and observed under the microscope. Red arrows indicate S. aureus bacteria. (TIF) [file pone.0063279.s001.tif]

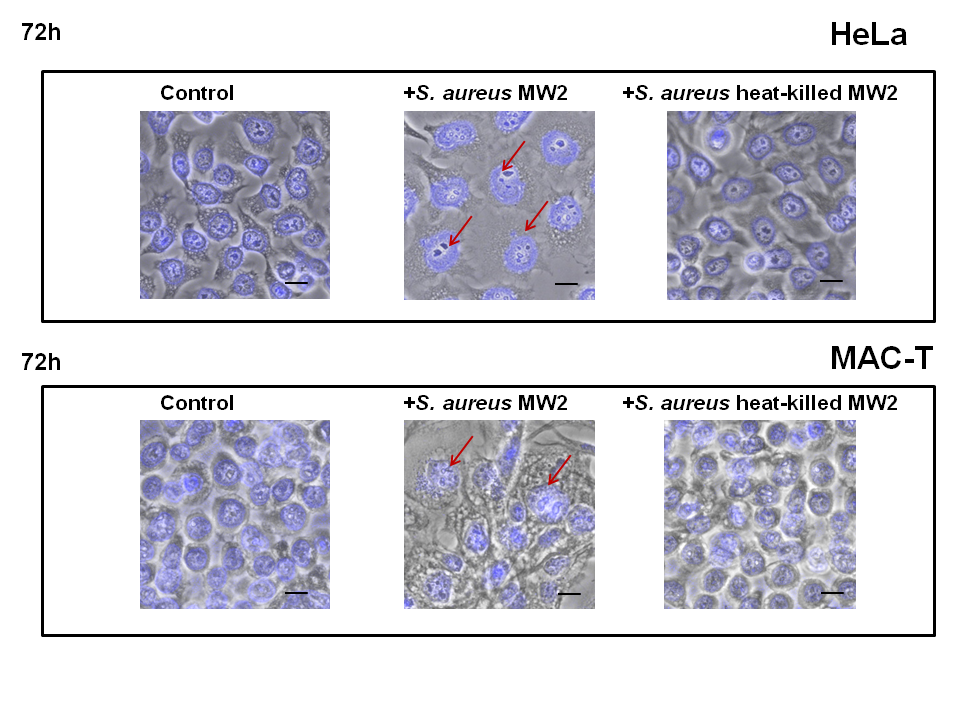

Supplement: Figure S3 — Exposure of eucaryotic cells to heat-killed S. aureus bacteria do not induce a cytopathic effect. Human HeLa or bovine MAC-T cells were exposed for 2 h to live or heat-killed S. aureus MW2 at MOI 20∶1 and further incubated for 72 h. The cells then were fixed, stained with DAPI, and observed using ×400 magnification. The merged image of phase contrast and DAPI-stained cells is presented. Red arrows indicate the enlarged cells in infected cell cultures. Microscopic observation revealed the enlargement of the cells exposed to live S. aureus bacteria. One representative experiment out of the three is shown. Scale bars: 10 µm. (TIF) [file pone.0063279.s003.tif]

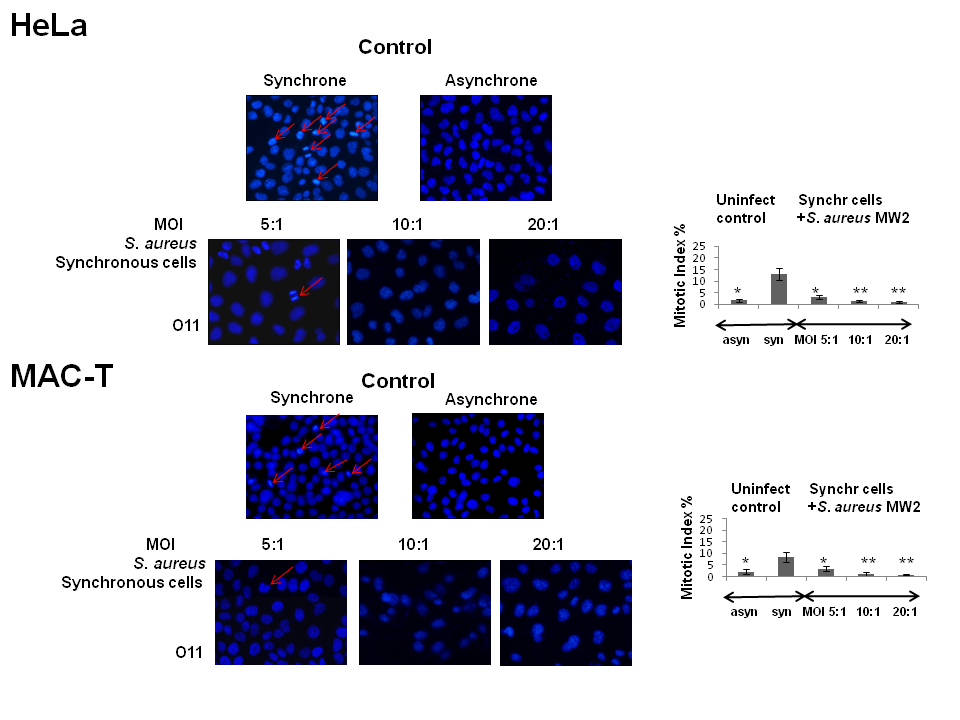

Supplement: Figure S5 — Decrease of the mitotic index in eucaryotic cells exposed to the O11 S. aureus strain. HeLa or MAC-T cells were synchronized by DTB and were then exposed to O11 S. aureus strains at MOIs ranging from 5∶1 to 20∶1 for 2 h, followed by incubation in cDMEM-Gent100 for 2 h, and then further incubated for 25 h. After centrifugation, the cells were fixed and stained with DAPI. Red arrows indicate the mitotic cells. The mitotic indexes in infected and in non-infected synchronous cells were evaluated by microscopic observation using ×400 magnification. Data are presented as mean +/− SD. The differences among the groups were assessed by ANOVA. (*) P-values <0.05 and (**) P-values <0.01 compared with control were considered to be significant. Tukey's Honestly Significant Difference test was applied for comparison of means between the groups. (TIF) [file pone.0063279.s005.tif]

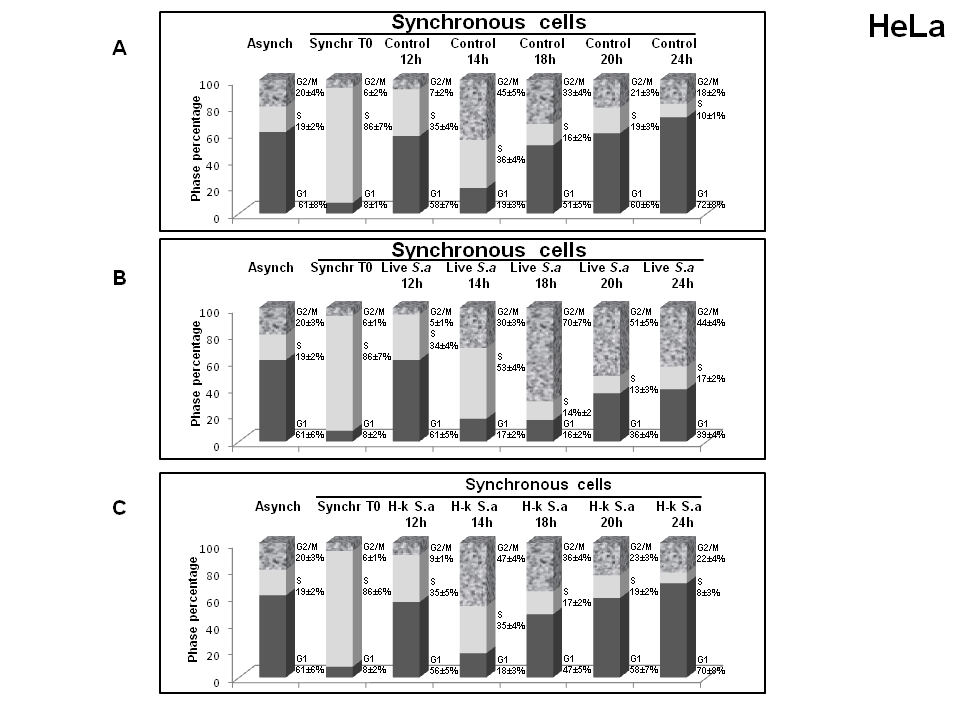

Supplement: Figure S6 — G2/M transition delay is induced by live S. aureus bacteria. HeLa cells were synchronized by DTB and were exposed to live or heat-killed S. aureus bacteria (MW2) at MOI 20∶1 for 2 h, followed by incubation in cDMEM-Gent100 for 2 h, and subsequent incubation for an additional 12 h, 14 h, 18 h, 20 h and 24 h. Detached cells were then combined with adherent cells and stained with PI. Cell cycle phases of PI-stained cells were monitored by FACS. The data were collected from 20,000 cells and analysis was performed with Cell Quest software. The number of cells in different phases is presented on the histograms. The values shown are those of a representative assay out of the four assays performed. (TIF) [file pone.0063279.s006.tif]
